# Synthesis of chemically cross-linked polyvinyl alcohol-co-poly (methacrylic acid) hydrogels by copolymerization; a potential graft-polymeric carrier for oral delivery of 5-fluorouracil

**DOI:** 10.1186/2008-2231-21-44

**Published:** 2013-05-30

**Authors:** Muhammad Usman Minhas, Mahmood Ahmad, Liaqat Ali, Muhammad Sohail

**Affiliations:** 1 Faculty of Pharmacy and Alternative Medicine, the Islamia University of Bahawalpur-63100, Punjab, Pakistan

**Keywords:** Polyvinyl alcohol, Methacrylic acid, Hydrogel, 5-Fluorouracil, pH-responsive

## Abstract

**Background of the Study:**

The propose of the present work was to develop chemically cross-linked polyvinyl alcohol-co-poly(methacrylic acid) hydrogel (PVA-MAA hydrogel) for pH responsive delivery of 5-Fluorouracil (5-FU).

**Methods:**

PVA based hydrogels were prepared by free radical copolymerization. PVA has been cross-linked chemically with monomer (methacrylic acid) in aqueous medium, cross-linking agent was ethylene glycol di-methacrylate (EGDMA) and benzoyl peroxide was added as reaction initiator. 5-FU was loaded as model drug. FTIR, XRD, TGA and DSC were performed for characterization of copolymer. Surface morphology was studied by SEM. pH sensitive properties were evaluated by swelling dynamics and equilibrium swelling ratio at low and higher pH.

**Results:**

FTIR, XRD, TGA and DSC studies confirmed the formation of new copolymer. Formulations with higher MAA contents showed maximum swelling at 7.4 pH. High drug loading and higher drug release has been observed at pH 7.4.

**Conclusions:**

The current study concludes that a stable copolymeric network of PVA was developed with MAA. The prepared hydrogels were highly pH responsive. This polymeric network could be a potential delivery system for colon targeting of 5-FU in colorectal cancers.

## Introduction

The term “hydrogel” is being considered for water insoluble polymeric network that has capacity to absorb large amount of water [1-5]. Synthetic or natural polymers, homopolymer or copolymer, are used to make three dimensional networks by molecular entanglements or by chemical crosslinking [6]. Physical or reversible hydrogels are synthesized by entanglements of polymer molecules or by hydrophobic interactions. Physical hydrogels can absorb the water but inhomogeneities or network defects may occur due to free chain ends or chain loops [7,8]. The network defects or loose aggregates in physical gels may cause the problem in drug loading and release formed by covalent crosslinking of polymers [9]. Chemical or permanent hydrogels are formed by covalent crosslinking of polymers [9]. Chemically cross-linked hydrogels do not change the shape under the ordinary pressure and erratic movements in GIT do not break the drug carrier systems. Therefore such stable system could be effective in site specific delivery of various agents.

First synthetic hydrogels of HEMA with EGDMA as cross-linker were prepared for biological use and later used for production of contact lenses [10]. Polyvinyl alcohol (PVA) based hydrogels have advantageous characteristics of good mechanical strength and high water retaining ability along with properties of biocompatibility, flexibility and can also be used as artificial soft tissue [11]. Mostly PVA hydrogels have been prepared by freezing and thawing cycles and by UV radiation cross-linking methods. PVA based hydrogels of different mechanical strengths can be produced by Freeze-thaw technique by controlling the freeze-thaw cycles but this would render the synthesis process time-consumption [12]. Various methods have been reported for physical cross-linking or non-chemical cross-linking methods of PVA graft polymers involving many processing steps and prolonged processes [13-16]. Chemical cross-linkers have employed in synthesis of number of non-PVA hydrogels. Previously PVA cross-linked membranes were synthesized using glutaraldehyde as cross-linking agent [17]. In present work, chemical cross-linking method has been developed for suitable polymer monomer ratio. The actual challenge was to study the reaction parameters, suitable polymer-monomer ratio and their optimization for temperature, time and concentrations of reactants to synthesize pH sensitive PVA graft MAA hydrogels. The study method could be considered a rapid and simple method for synthesis of PVA-co-poly(methacrylic acid) hydrogels by chemical cross-linking with EGDMA. A monomer MAA was used to impart pH sensitive characteristics. The study was undertaken to synthesize and characterize the pH sensitive chemically cross-linked PVA hydrogels and to introduce a promising delivery system for colonic delivery of 5-fluorouracil in colorectal cancer (5-FU is a first line anticancer drug in colorectal cancer therapy). Chemical structures of polymer, monomer and presumptive cross-linked hydrogel structure as well as their abbreviations have been listed in Table 1.

**Table 1:** Chemical structures of Polymer, monomer and possible cross-linked structure of hydrogels

**Monomer/Polymer**	**Abbreviation**	**Chemical structure**
Methacrylic acid	MAA	
Polyvinyl alcohol	PVA	
Ethylene glycol di-methacrylate	EGDMA	
Cross-linked polyvinyl-co-poly(Methacrylic acid) hydrogels	PVA-co-poly (MAA)	

## Materials and methods

### Chemicals

5-Fluorouracil was obtained as a kind gift from Pharmedic Laboratories (Pvt.) Ltd. Lahore, Pakistan. Polyvinyl alcohol (PVA), methacrylic acid (MAA), ethylene glycol dimethacrylate (EGDMA), benzoyl peroxide were purchased from Sigma Aldrich, UK., Deionized distilled water was obtained from our laboratory.

### Synthesis of PVA-co-polyMAA hydrogels

Free radical copolymerization method was adopted and various formulations were prepared that have been presented in Table 2. A specific quantity of PVA was weighed and dissolved in water by continuous stirring at 800 rpm and heating of the reaction mixture was maintained at 90°C, until a transparent solution was obtained. The reaction mixture was purged under the nitrogen stream for 30 min to exclude the dissolved oxygen. A separate solution was prepared in which benzoyl peroxide as initiator was dissolved in weighed amount of MAA. The benzoyl peroxide-MAA mixture was added slowly (drop wise) in PVA solution at 50°C. Finally ethylene glycol diamine methacrylate (EGDMA) was added (0.5 mol% of monomer) to the reaction mixture (Table 2). The resultant reaction mixture was carefully transferred to glass tubes of same dimensions and heated in water bath at 55°C for 4 hours, 60°C for 6 hours and 65°C for 12 hours. After this treatment, all tubes were cooled to room temperature and cylindrical hydrogels were cut into small discs (8 mm in length). These discs were washed with ethanol-water (70:30) to remove un-reacted monomers and catalyst, until no change in pH of solvents mixture before and after washing of discs. Initially the discs were dried in laminar flow air for 24 h and then in vacuum oven at 40°C for one week.

**Table 2:** Formulations of PVA-co-poly(MAA) hydrogels

**Formulation code**	**Polyvinyl alcohol (g/100 g)**	**Methacrylic acid (g/100 g)**	**EGDMA mol% of monomer**
PVH-1	0.500	20.0	0.10
PVH-2	1.00	20.0	0.10
PVH-3	2.00	20.0	0.10
PVH-4	2.00	20.0	0.10
PVH-5	2.00	30.0	0.10
PVH-6	2.00	40.0	0.10
PVH-7	1.00	20.0	0.20
PVH-8	1.00	20.0	0.25
PVH-9	1.00	20.0	0.30

### Characterization

#### FT-IR

The FT-IR spectra of pure PVA and MAA were recorded. Samples were thoroughly ground and analyzed by attenuated total reflectance ATR-FTIR (Shimadzu, Germany) in range of 4000–650 cm^-1^. All the hydrogel formulations were examined by FT-IR.

#### TGA and DSC

Thermal analysis was performed by thermogravimetric analysis (TGA) of TA instruments Q5000 series Thermal Analysis System (TA instruments,WestSussex, UK) and differential scanning calorimetry (DSC) of TA instruments Q2000 Series Thermal Analysis system (TA Instrument WestSussex, UK). The hydrogel samples were ground and passed through mesh 40. For TGA, amount between 0.5-5 mg was placed in an open pan (platinum 100 μl) attached to a microbalance .The samples were heated at 20°C/min from 25-500°C under dry nitrogen at standard mode with ramp test type. All the measurements were made in triplicate. For DSC, samples of PVA, MAA and formulations (0.5-3 mg) were precisely weighed into an aluminum pan onto which aluminum lid with a central pierced hole was crimped. The samples were then scanned under a stream of nitrogen gas from 0-400°C using heating rate 20°C/min.

#### PXRD

Bruker D-8 powder diffractometer (Bruker Kahlsruhl, Germany) was used to record the XRD pattern, at room temperature. Powdered samples were filled on to plastic sample holder and smoothing the surface with a glass slide. Samples were scanned over range 5-50° 2θ at a rate of 1^º^ 2θ/min using a copper Kα radiation source with a wavelength of 1.542 Å and 1 mm slits.

#### Morphology of networks

Surface morphology of hydrogels was investigated using scanning electron microscopy (SEM) by a Quanta 400 SEM (FEI Company, Cambridge, UK). Completely dried discs of hydrogels were cut to optimum sizes to fix on a double-adhesive tape stuck to an aluminum stub. The stubs were coated with gold to a thickness of ~300 Å under an argon atmosphere using a gold sputter module in a high-vacuum evaporator. The coated samples were randomly scanned and photomicrographs were recorded to reveal surface morphology.

### Equilibrium swelling ratio

Swelling studies were performed to evaluate the pH sensitivity of the hydrogels. Dried discs of hydrogels were weighed and immersed in 0.1 M HCl pH 1.2 and in phosphate buffer solutions of 5.8, 7.4 at 37°C. The samples were removed at specific intervals and weighed after removing excess of water by blotting with filter paper. The degree of swelling and equilibrium water contents were calculated using Eq. 1 and 2 [18].

(1) $$Q=\frac{M_{s}}{M_{d}}$$

(2) $$\mathrm{EWC\%}=\frac{M_{\mathrm{eq}\_}M_{d}}{M_{d}}\times 100$$

Where *M*_*s*_ indicates mass of swelling at predetermined time interval, *M*_*eq*_ is weight at equilibrium swelling and *M*_*d*_ represents the weight of dry gel before initiation of swelling experiments.

### Drug loading and release studies

5-Fluorouracil (5-FU) was loaded in hydrogels by absorption method [17,19,20] as model drug by diffusion method. Dried circle PVA-co-MAA hydrogels discs (8 mm) were immersed into 100 ml 5-FU solution (1.0%) in phosphate buffer of pH 7.4 for 72 hours at room temperature. Higher pH and solvent was selected in which drug showed maximum solubility and higher swelling. The discs were immediately washed with distilled water and first dried at room temperature and then placed in oven at 40°C.

Percentage of drug loading was assessed by extracting the weighed amount of polymer with same solvent used for drug loading. Each time 25 ml of fresh buffer solution was used to extract the drug from discs. Extraction was repeated until no drug found in solution. Drug contents were determined by preparing calibration curve of 5-FU dilutions in phosphate buffer using UV–vis-spectrophotometer (UV-1601Shimadzu). The sample was scanned first to determine the λ _max_ that was found 266 nm.

Drug release was investigated at low and high pH values to confirm the pH dependant delivery of 5-FU from PVA/MAA hydrogel network. Drug loaded disks were evaluated for 5-FU release in 900 ml solutions of pH 1.2 and 7.4 in USP dissolution apparatus-II at 37 ± 0.5°C. These samples were analysed at 266 nm using UV–vis-spectrophotometer (UV-1601Shimadzu).

## Results and discussion

### Physical appearance of hydrogels

**Figure 1:**
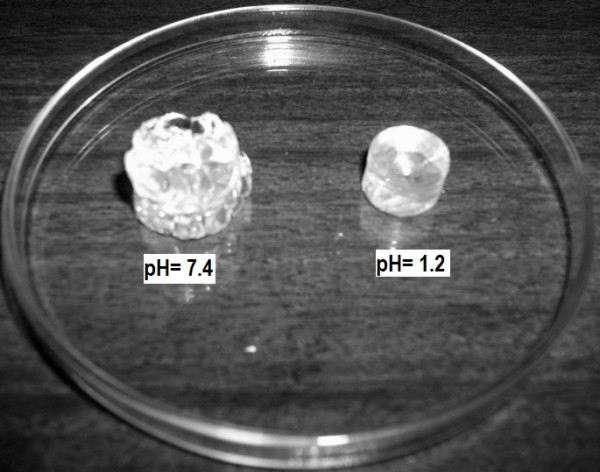
PVA Hydrogels after swelling at pH 1.2 and 7.4.

Polymerization of PVA with MAA after the crosslinking formed stable polymeric networks. Gels with higher MAA concentration revealed glass like transparent appearance while gels with low MAA contents showed milky white characteristics. An excellent mechanical strength was observed in gelled copolymer that retained the shape even after swelling. Hydrogels with high MAA ratio showed higher mechanical strength than hydrogels with higher polymer (PVA) contents. Discs in swelled form have been shown in Figure 1.

### Structure analysis

**Figure 2:**
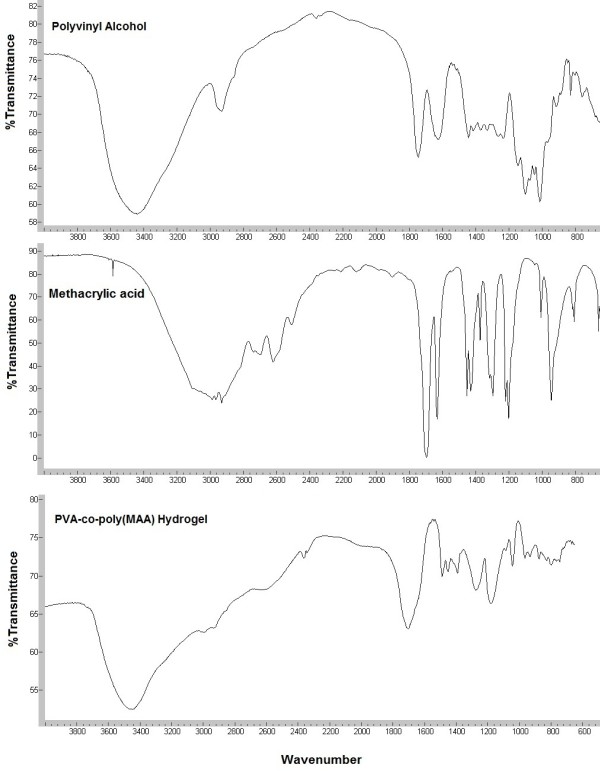
FTIR spectra of PVA, MAA and PVA-co-MAA hydrogels.

FT-IR spectra of PVA, MAA and PVA-co-poly(MAA) hydrogel have been shown in Figure 2. PVA spectra showed broad absorption at region of 3400 cm^-1^ indicating the presence O-H stretching vibrations. Peak at 2940 cm^-1^ is related to presence of –CH_2_ groups. A band at region of 1449 cm^-1^ showed –OH deformation. The FT-IR spectrum of methacrylic acid indicates peak at 2929 cm^-1^that indicates the presence of methyl C–H asymmetric stretching. Band range in 1725–1700 cm^-1^ is assigned for carboxylic acid (1699 cm^-1^ indicate carboxylic acid groups) and the peak at 1633 cm^-1^ shows the C=C stretching vibrations.

The spectrum from cross-linked hydrogel formulations showed different peaks from the parent components (polyvinyl alcohol and methacrylic acid). Broad absorption at 3467 cm^-1^ showed –OH stretching and a absorption at region of 1700 cm^-1^ revealed the presence of carbonyl group that was not present in pure PVA spectrum which indicates the esterification between PVA and MAA.

**Figure 3:**
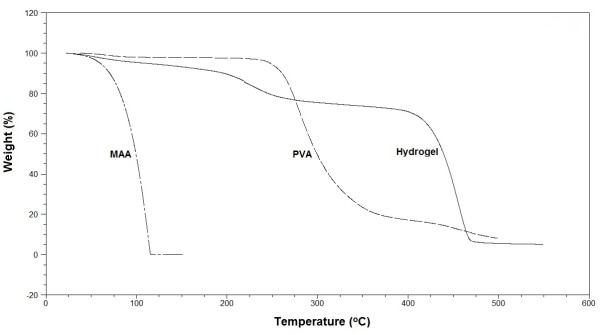
TGA of PVA, MAA and PVA-co-MAA hydrogels.

**Figure 4:**
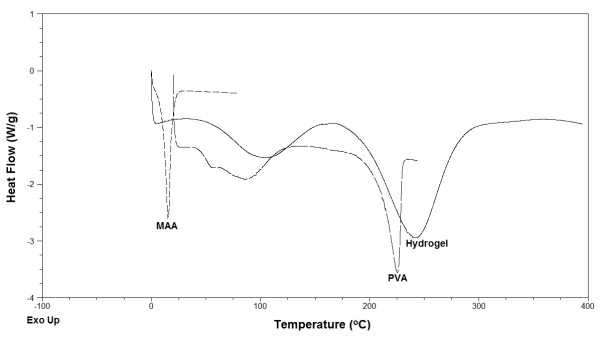
DSC of PVA, MAA and PVA-co-MAA hydrogels.

TGA and DSC measurements were performed for the pure PVA, MAA and PVA-MAA hydrogels, shown in Figures 3 and 4. Thermogram analysis was performed to determine the thermal stability. Initial loss in mass was due the water loss. TGA curves showed that PVA was stable up to 250°C and decomposition occur above the 265°C. Complete loss of pure MAA occur above 100°C. However PVA-MAA hydrogel revealed first endothermic peak from 225°C to 325°C and decomposition curve from 400°C to 475°C. PVA-co-MAA showed higher thermal stability pattern, it indicates that cross-linking between the PVA and MAA increase the thermal stability of PVA. This showed that the formed hydrogels could be processed at reasonably higher temperature than its individual components (PVA, MAA and EGDMA).

DSC endothermic peaks of pure PVA and MAA were different from the cross-linked PVA-MAA macromolecule. The DSC endothermic peak of PVA at 85°C can be attributed as Tg and decomposition start at 200°C that is melting temperature. Complete mass loss was observed for MAA at 25°C. DSC thermograms of PVA-MAA hydrogel formulations showed two major endothermic peaks, mass loss at 100°C could be attributed to water loss from preparation. However peak at 240°C showed the decomposition of cross-linked polymer-monomer networks, similar reponse by TGA thermogram has been recorded that indicates the decomposition range from 180 to 325°C. TGA and DSC thermograms indicate different thermal pattern of pure PVA and MAA from prepared hydrogels. Cross-linking between PVA and MAA increases the thermal stability and these cross-linked matrices could provide a good delivering capacity for various types of drug molecules.

**Figure 5:**
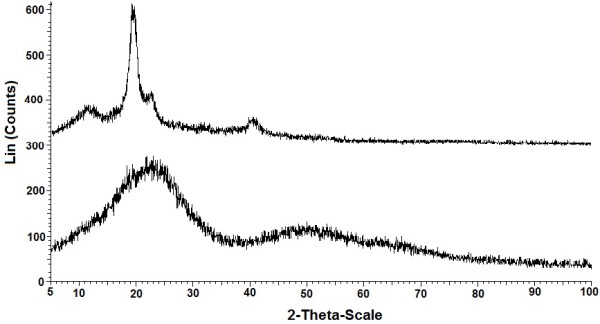
XRD patterns of pure PVA and PVA-co-MAA hydrogels.

PXRD pattern of pure PVA and PVA-MAA hydrogels was recorded that has been shown in Figure 5. A sharp peak at 2θ=20.1° is the characteristic of PVA. PVA-MAA hydrogel showed different XRD pattern from the pure PVA that confirmed the formation of a new polymer. The peak at 2θ=20.1° is weekend significantly and another broad peak also appeared at 2θ=50.0°. The XRD pattern of PVA-MAA hydrogels showed decrease in the crystallinity. The cross-linking between the PVA and MAA decrease the crystalline behavior of PVA.

**Figure 6:**
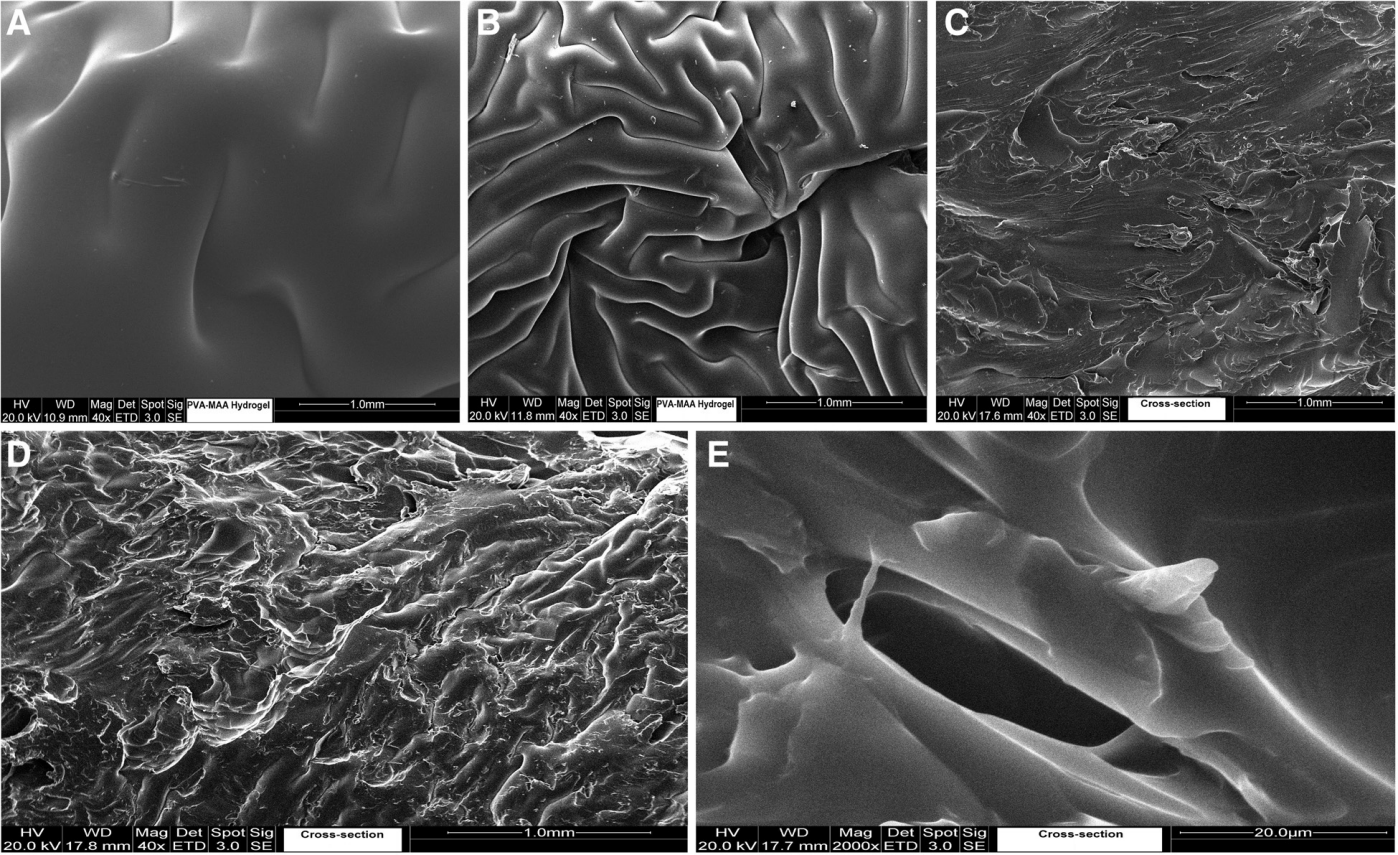
SEM micrographs of PVA-co-MAA hydrogels; (A) & (B) intact surface, (C) (D) & (E) cross-section.

Morphological studies were performed for intact surface studies of hydrogels and cross-sectional part by SEM. SEM micrographs revealed that hydrogel formulations with low polymer contents but high monomer contents indicates comparatively smooth outer surface while hydrogels with higher polymer concentration showed wavy surface. Surface morphology of intact discs and cross-sections are presented in Figure 6, hydrogels with higher monomer (MAA) contents revealed less wavy surface than Hydrogel discs with higher polymer ratio. Cross-sectional images of hydrogels with low polymer contents revealed large pores and dense areas (Figure 6). However smaller but number of pores were observed in cross-sectional images of hydrogels with high polymer. Generally a porous structure has been observed in morphological studies.

### Effect of polymer, monomer and cross-linker on swelling

**Figure 7:**
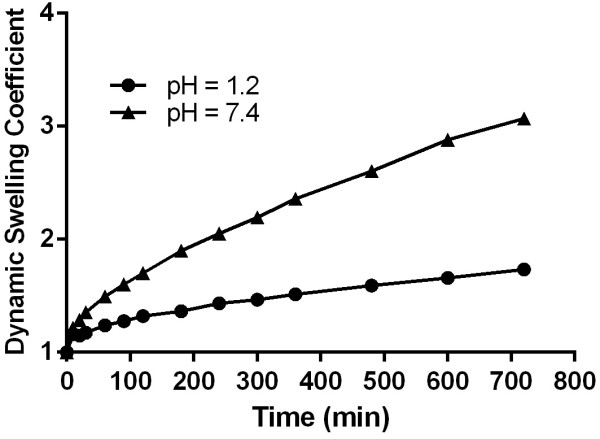
Effect of PVA, MAA and EGDMA on swelling index.

**Figure 8:**
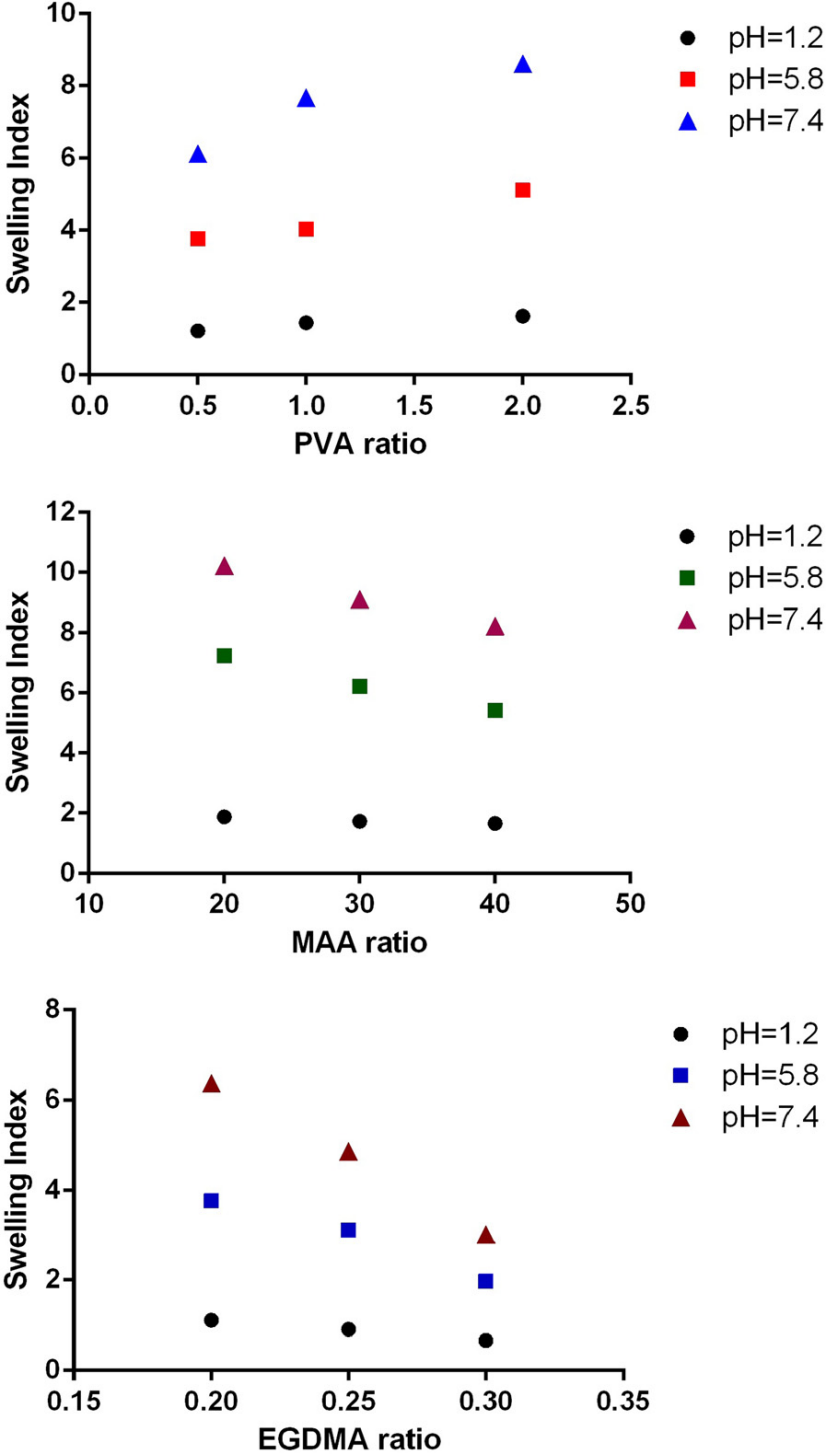
Dynamic swelling characteristics of PVA-MAA hydrogels (PVH-3).

Various formulations were prepared with varying the polymer, monomer and cross-linker ratio. Three sets of sample series were prepared to evaluate the effect of polymer (PVA), monomer (MAA) and cross-linker (EGDMA) contents on dynamic swelling of hydrogels. Dynamic swelling was studied to evaluate the swelling with respect to time and representative swelling has been shown in Figure 7. Formulation series PVAH-1 to PVAH-3 with increasing concentration of polymer (PVA), PVAH-4 to PVAH-6 with increasing contents of monomer (Methacrylic acid) and PVAH-7 to PVAH-9 with increasing the cross-linker’s concentration were prepared. In prepared hydrogels pH dependent swelling was observed that could be attributed to ionizable functional groups. PVA contain high hydroxyl groups that make this polymer highly interactive with water. Large pendent carboxylic groups associated with methacrylic acid made the coploymeric system a pH responsive that could be observed from swelling characteristics. At high pH these carboxylic groups ionized and repel each other. The repulsive forces impart the swelling properties to gels. Effect of polymer, monomer and cross-linker on swelling (equilibrium swelling index) at various pH has been presented in Figure 8. Dynamic swelling was studied to evaluate the swelling with respect to time and representative swelling has been shown in Figure 7. Hydrogels with increasing ratio of polymer (PVAH-1 to PVAH-3) did not show significant difference in dynamic swelling up to 12 hrs from each other.

### Drug loading and release studies

**Figure 9:**
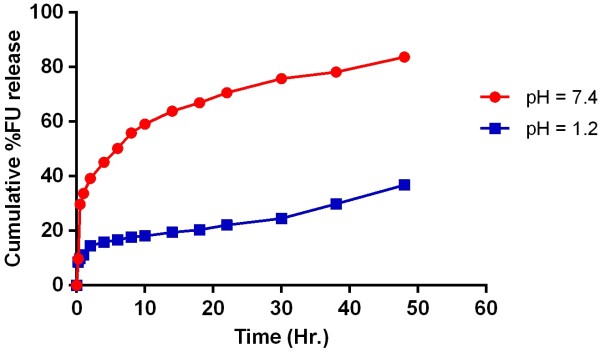
FU release studies from PVA-MAA hydrogels (PVH-3) at pH 1.2 and 7.4 up to 48 hrs.

5-Fluorouracil was loaded as model drug to evaluate its delivery against the pH stimuli. Drug loading was found 0.115 ± 0.02 mg of FU/mg of hydrogel. Higher drug loading was observed in discs that showed higher swelling. Percent release studies of 5FU at pH 1.2 and 7.4 have been presented in Figure 9. Low drug released at pH 1.2 but higher at pH 7.4. The prepared hydrogels showed drug release up to 48 hours in phosphate buffer of pH 7.4. The pH responsive delivery of this anticancer drug could be highly important because the prepared hydrogels showed higher 5-FU at high pH for longer period of time (approximately 80% during 48 hours. Percentage of drug release has also been presented in Table 3 to evaluate the effect of ratio (up to 24 hours).

**Table 3:** Effect of reaction variables on drug loading and percent release

**Formulation code**	**5FU loading mg/0.5 g of dry gel**	**% release of 5FU up to 24 hrs.**		
		**pH 1.2**	**pH 5.8**	**pH 7.4**
PVH-1	72.62	4.66	32.2	63.67
PVH-2	79.22	5.19	39.3	68.23
PVH-3	88.81	6.01	44.1	73.29
PVH-4	89.65	7.21	51.1	78.55
PVH-5	84.44	6.22	47.3	74.19
PVH-6	75.23	5.98	41.2	70.37
PVH-7	81.71	6.63	34.4	70.32
PVH-8	72.91	5.32	28.8	63.35
PVH-9	67.26	3.81	22.2	56.44

## Conclusions

Chemical cross-linking between polyvinyl alcohol and methacrylic acid modified the characteristics of individual components and formed a pH responsive co-polymeric matrix system. The chemical cross-linking of PVA by EGDMA impart a good strength, very low water absorption at low pH and high at high pH. The prepared hydrogels showed a pH responsive behavior as well as higher drug release at high pH. 5-FU has been loaded as model drug that is being used in cancer chemotherapy but intravenously. It can be concluded that a promising chemically cross-linked pH responsive co-polymeric matrix delivery would be highly effective in colorectal cancer therapies.

## Abbreviations

PVA: Polyvinyl alcohol; MAA: Methacrylic acid; 5-FU: 5-Fluorouracil.

## Competing interests

All authors declared that they have no competing interests.

## Authors’ contributions

All the authors have substantial contribution in completion of this study. MUM prepared the formulations, analyzed and wrote the manuscript. MA supervised the study and reviewed the manuscript. LA and MS participated in experimental work. All authors read and approved the final manuscript.
